# Cellulolytic and hemicellulolytic capacity of *Acetivibrio clariflavus*

**DOI:** 10.1007/s00253-025-13471-9

**Published:** 2025-04-28

**Authors:** Katarína Šuchová, Vladimír Puchart

**Affiliations:** Institute of Chemistry, Slovak Academy of Sciences, Dúbravská cesta 9, 845 38 Bratislava, Slovakia

**Keywords:** *Acetivibrio clariflavus*, Cellulosome, Cellulose, Hemicellulose, Xylan, Glycoside hydrolase, Carbohydrate esterase, Polysaccharide lyase

## Abstract

**Abstract:**

Lignocellulosic biomass represents the largest available renewable source of carbon. It is a recalcitrant mixture of different polysaccharides and lignin. For its complete degradation, a large set of enzymes capable of cleaving its individual components is required. Several anaerobic bacteria produce high-molecular-weight multienzyme complexes called cellulosomes where the enzymes work in synergy for efficient degradation of the complex plant material. One of the anaerobic thermophilic cellulosome-forming bacteria is *Acetivibrio clariflavus*. *Acetivibrio clariflavus* was found to be one of the most abundant lignocellulose-solubilizing bacteria in various plant decaying environments. It produces sophisticated cellulosomal complex that is most similar to mesophilic *Acetivibrio cellulolyticus* cellulosome. In comparison with other anaerobic cellulosome-forming bacteria *A*. *cellulolyticus* and *Acetivibrio thermocellus*, *A. clariflavus* possesses lower number of cellulolytic enzymes. However, it is significantly better equipped for a degradation of hemicellulose, particularly xylan. Some strains, e.g., 4 - 2a, were also shown to utilize xylose. Efficient saccharification of plant biomass makes *A. clariflavus* a promising candidate for various biotechnological applications including biofuel production.

**Key points:**

*• Acetivibrio clariflavus is anaerobic thermophilic cellulosome–forming bacterium.*

*• Its cellulosomes target mostly cellulose and hemicellulose, in particular xylan.*

*• The strains share most of xylanolytic enzymes but differ in xylose utilization.*

**Supplementary Information:**

The online version contains supplementary material available at 10.1007/s00253-025-13471-9.

## Introduction

Thermophilic anaerobic bacteria with lignocellulolytic capacity are members of microbial consortia thriving in environments where lignocellulosic material is degraded at higher temperatures. These conditions select microorganisms capable of producing a range of thermostable enzymes that are expected to work under extreme conditions required in many biotechnological applications including pulp and paper industry, biomass degradation, or biofuel production. The best studied lignocellulolytic anaerobic thermophilic bacterium is *Acetivibrio thermocellus* (synonyms *Clostridium thermocellum*, *Ruminiclostridium thermocellum*, *Hungateiclostridium thermocellum*) producing cellulosomes with high cellulolytic activity but unable to grow on xylan and pentose sugars (Zverlov and Schwarz 2008). On the contrary, *Thermoclostridium (Clostridium) stercorarium* produces a set of hemicellulolytic enzymes in a free form and ferments a wide range of sugars including pentoses (Broeker et al 2018). Extremely thermophilic cellulolytic and xylanolytic bacteria are from the genus *Caldicellulosiruptor* (*C. saccharolyticus*, *C. bescii, C. obsidiansis*) (Singh et al. 2021). Another efficient lignocellulose degrader is *Acetivibrio clariflavus*, which was found to be one of the most abundant lignocellulose-solubilizing bacteria in various environments including self-heated biocompost, anaerobic sludge, or garbage slurry (Shiratori et al. 2009; Izquierdo et al. 2010; Liang et al. 2018). Several strains were isolated, all having high cellulolytic capacity but differing in their ability to utilize xylan and xylose. In this review, we give a comprehensive overview of cellulolytic and hemicellulolytic capacity of *A. clariflavus* strains. We summarize information about few (hemi)cellulolytic enzymes, which were so far biochemically characterized, and we suggest the specificity of others based on similarity to described enzymes from other microorganisms, mostly clostridia.

## *Acetivibrio clariflavus* isolation and characterization

*Acetivibrio clariflavus* belongs to the family *Oscillospiraceae* within the class *Clostridia*. Originally, it was named *Clostridium clariflavum*, but in 2018, it was reclassified and renamed to *Hungateiclostridium clariflavum* (Zhang et al. 2018) and in 2019 to *Acetivibrio clariflavus* (Tindall 2019).

*Acetivibrio clariflavus* (deposited as type strain EBR45 = DSM 19732 = NBRC 101661) was first isolated from anaerobic sludge of a cellulose-degrading methanogenic bioreactor (Shiratori et al. 2009). It was characterized as a rod-shaped anaerobic, thermophilic, spore-, and cellulosome-forming bacterium growing in non-motile flagellated rods utilizing well cellulose and cellobiose as sole carbon sources but not starch and monosaccharides glucose, xylose, and arabinose (Shiratori et al 2009).

*Acetivibrio clariflavus* strain CL-1 was isolated as a cellulolytic bacterium from a thermophilic methanogenic reactor used for a degradation of garbage slurry (Sasaki et al. 2012). The closest relative of the strain CL-1 was found to be *A. clariflavus* type strain DSM 19732 (99.9% sequence similarity).

The strains identified as *A. clariflavus* were also found in several other cellulolytic environments. *Acetivibrio clariflavus* was recognized as the key functional microbe of a cellulolytic microbial consortium grown in fermentation medium with filter paper as carbon source (Zhang et al. 2013). It was found to be well represented within biogas microbiomes (Maus et al. 2017) and was one of the most abundant lignocellulose-solubilizing and xylose-fermenting bacterium during a switchgrass fermentation (Liang et al. 2018). *Acetivibrio clariflavus* DSM 19732 was used for carbohydrate solubilization of mid-season harvested switchgrass, where around 50% conversion of glucan and xylan was achieved after 5 days (Paye et al. 2016). A thermophilic microbial consortium RSX was constructed with *A. clariflavus* DSM 19732 as one of the key bacteria, which could efficiently degrade the waste cassava residue from alcohol fermentation (Zhang et al. 2011). A co-culture of *A. clariflavus* CL-1 and the hydrogenotrophic methanogen *Methanothermobacter thermautotrophicus* ΔH increased the degradation efficiency of cellulose and cell density of the strain CL-1 as well as a production of acetate (Sasaki et al. 2012).

*Acetivibrio clariflavus* was also identified as predominant organism in one of cellulolytic enrichment cultures obtained when thermophilic compost was used as an inoculum (Izquierdo et al. 2010). Two strains (4-1 and 4-2a) were subsequently isolated from self-heated biocompost (Sizova et al. 2011). The 16S rRNA gene sequence similarities for strains 4-1 and 4-2a were 100% with respect to each other and 99.7% with respect to previously isolated *A. clariflavus* DSM 19732. Both isolates showed high cellulase and xylanase activity during the growth on cellulose or xylan. Unlike the strain DSM 19732, the strains 4-1 and 4-2a utilized also xylose and pretreated wood, however, the growth on xylose was slow (Sizova et al. 2011). The utilization of pentose sugars is a feature that distinguishes *A. clariflavus* strains 4-1 and 4-2a from another cellulolytic thermotolerant and well-characterized bacterium *Acetivibrio thermocellus*.

The differences in the ability of the *A. clariflavus* stains DSM 19732 and 4-2a to utilize xylan and xylose led to a comparison of assimilation of xylose, xylooligosaccharides (XOs, xylobiose Xyl_2_, xylotriose Xyl_3_, and xylotetraose Xyl_4_), birchwood xylan, and unpretreated switchgrass by both strains as well as the model thermophile *A. thermocellus* ATCC 27405 (Izquierdo et al. 2014). The three bacteria behaved differently on all these carbon sources. Xylan was hydrolyzed by *A. thermocellus* to XOs, which remained in the medium, but no xylose or growth of *A. thermocellus* was observed. *Acetivibrio clariflavus* DSM 19732 initially produced XOs, which were quickly hydrolyzed to xylose, but the microorganism did not grow. In opposite, *A. clariflavus* 4-2a was able to grow on xylan. XOs appeared early in the fermentation process but were quickly broken down to xylose, which was slowly utilized and partly remained in the medium after 144 h of incubation. The growth was accompanied with acetate and formate formation that also served as evidence of fermentation (Izquierdo et al. 2014). When XOs were used as the carbon source, the results were similar—*A. clariflavus* 4-2a was able to hydrolyze them to xylose and to grow, while the strain DSM 19732 only cleaved them to xylose, which was not further utilized. Accordingly, xylose (2.5 g/L) was consumed only by *A. clariflavus* 4-2a, although its utilization was slow, taking approximately 120 h. *Acetivibrio clariflavus* 4- 2a was also the strain most efficiently solubilizing the switchgrass (58.8%). All three microbial strains preferentially utilized loosely associated hemicellulosic xylans in switchgrass biomass, *A. clariflavus* strains being more efficient in terms of xylan solubilization (Izquierdo et al. 2014).

In 2012, complete genome sequence of *A. clariflavus* DSM 19732 was published (Izquierdo et al. 2012). The phylogenetic relationship of the 16S rRNA gene of *A. clariflavus* DSM 19732 showed at least 96.6% sequence homology with other thermophilic cellulolytic bacteria *Clostridium straminisolvens* and *A. thermocellus*. The genome of *A. clariflavus* DSM 19732 comprise one circular chromosome of 4,897,678 bp in length with 35.6% GC content and is predicted to harbor 4242 coding gene sequences. Comparative genome analysis of *A. clariflavus* DSM 19732 and *A. thermocellus* ATCC 27405 indicated that ~ 70% of glycoside hydrolases (GHs) are similar in both genomes. While cellulolytic enzymes are similarly distributed in these two species, *A. clariflavus* DSM 19732 has a higher number of genes coding for xylanolytic enzymes. In addition, putative xylulose kinase (Clocl_2440) and xylose epimerase (Clocl_2439) genes have been identified in the *A. clariflavus* DSM 19732 genome within a gene cluster encoding hemicellulose-active enzymes, however, a gene coding for xylose isomerase was not found. The absence of this gene explains the inability of the DSM 19732 strain to utilize xylose and xylans (Izquierdo et al. 2012). In contrast, the genome sequence of the *A. clariflavus* 4-2a revealed the presence of the xylose isomerase gene along with the xylulose kinase gene in a genomic island not present in the type strain DSM 19732 (Rooney et al. 2015). The inventory of glycoside hydrolases is almost identical for both strains, except of an additional putative GH3 β-glucosidase/xylosidase identified in the same genomic island of the strain 4-2a (Rooney et al. 2015).

## Cellulosomes produced by *A. clariflavus*

**Fig. 1:**
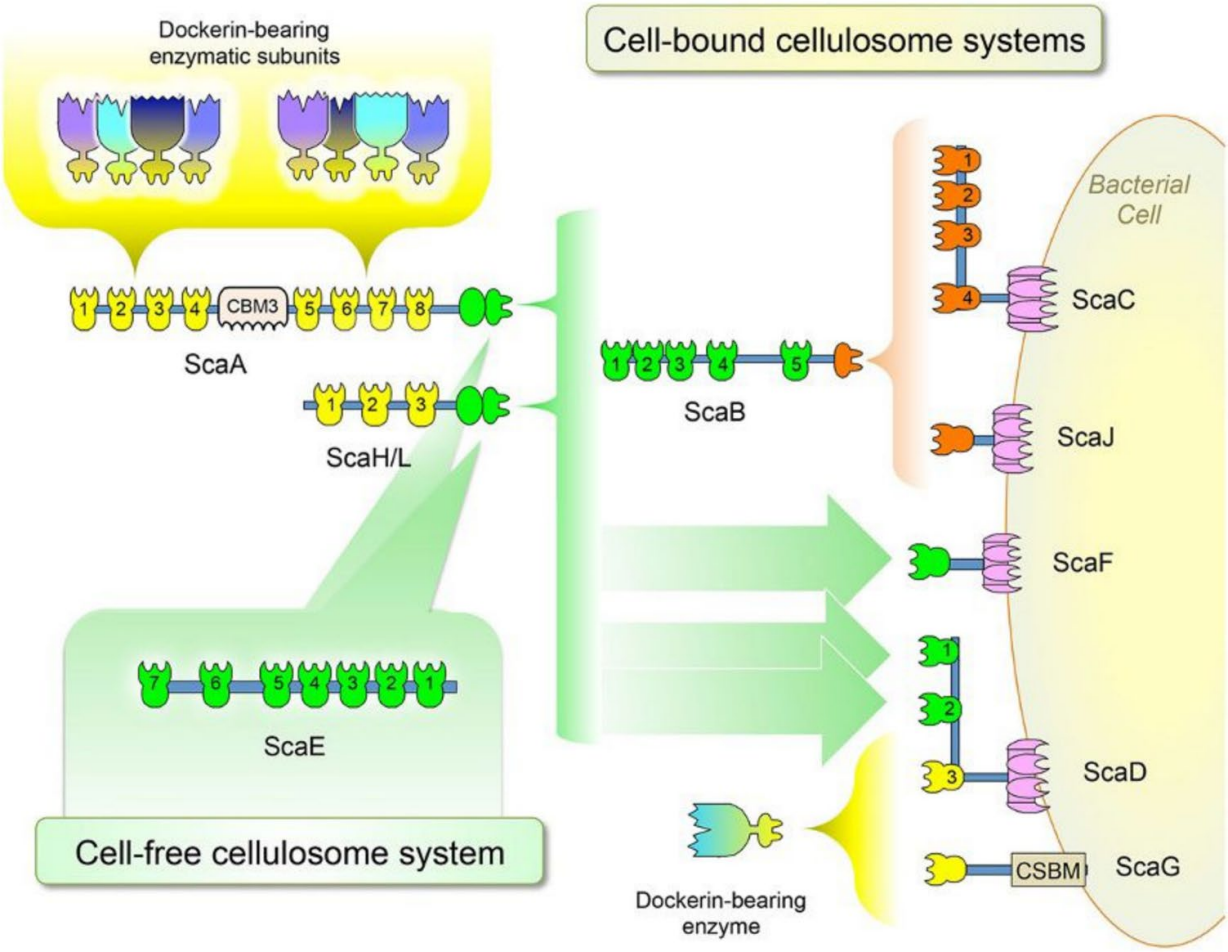
Proposed architectures for cell-bound and cell-free cellulosome assembly in *Acetivibrio clariflavus*. The scheme shows the possible interactions among scaffoldins and enzymatic modules. Four potential cell-anchored cellulosomal complexes are represented. Two of the complexes employ the adaptor protein ScaB to join the cell-anchored scaffoldins (ScaC and ScaJ) to the primary enzyme-integrating scaffoldins (ScaA and ScaH/L) via the type II cohesins (green) of ScaB and C-terminal XDocs of ScaA and ScaH/L (green). The type II cohesins of ScaD (green cohesins 1 and 2) and ScaF (single green cohesin) are also cell-anchored scaffoldins that bind directly ScaA or ScaH/L. The type I cohesins of ScaG (single yellow cohesin) and ScaD (yellow cohesin 3) interact with type I dockerins of dockerin-bearing enzymes. ScaG is suspected to be a cell-anchored scaffoldin. ScaE has seven type II cohesins (green) which are able to bind seven XDoc modules, thereby creating a large, cell-free cellulosomal complex. CSBM, cell surface-binding module. The figure is reproduced from Artzi et al. (2014)

In order to efficiently degrade cellulosic substrates, several anaerobic bacteria including *A. clariflavus* produce high-molecular-weight multienzyme complexes called cellulosomes. They consist of two types of proteins—scaffoldins and enzymes. The scaffoldins are mostly non-catalytic proteins bearing cohesion domains, which are recognized by dockerin domains linked to a variety of carbohydrate active enzymes. In this way, the large multi-enzyme cellulosome complexes can be formed and released into the extracellular medium or attached to the cell surface. The bioinformatic analysis of cellulosomic system of *A. clariflavus* DSM 19732 revealed the presence of 49 cohesin modules distributed in 13 different scaffoldins ScaA to ScaO and 79 dockerin-containing proteins (Artzi et al. 2014). All 13 scaffoldins showed similarity to the proposed cellulosome system of *Acetivibrio cellulolyticus* (synonym: *Hungateiclostridium cellulolyticum*) consisting of 16 scaffoldins, which is twice the number of scaffoldins in *A. thermocellus* (eight scaffoldins) (Artzi et al. 2017). ScaE is a part of cell-free cellulosome and consists exclusively of seven type II cohesins, which are closely related to the seven cohesin modules of ScaE from *A. cellulolyticus* and Cthe_0736 from *A. thermocellus* (Artzi et al. 2014; Dassa et al. 2012). ScaC, ScaD, ScaJ, ScaF, and ScaG of all three bacteria contain cell-anchoring domains (mostly S-layer homology (SLH) domains). ScaC and ScaJ of *A. clariflavus* employ the adaptor protein ScaB to join them to the primary enzyme-integrating scaffoldins ScaA and ScaH/L (Fig. 1). A fully occupied system of ScaC-ScaB-ScaA would theoretically accommodate 160 enzymatic units, which represent the largest cellulosomal complex yet discovered (Artzi et al. 2014). Similar system in *A. cellulolyticus* can integrate up to 96 enzymatic units. Homolog of ScaB in *A. thermocellus* cellulosome system does not serve as an adaptor protein and is attached directly to the cell. Highly structured cellulosome complex of *A. thermocellus* contains up to 63 enzymes (Artzi et al. 2017). The cohesins I and CBM2 modules of three *A. clariflavus* scaffoldins ScaM, ScaM(a), and ScaM(b) are phylogenetically related to the *A. cellulolyticus* ScaM. This type of scaffoldins is not found in *A. thermocellus*.

The dockerin-containing proteins include 40 enzymes from 15 glycoside hydrolase (GH) families, 14 carbohydrate esterases (CEs), and three polysaccharide lyases (PLs). Some of the enzymes contain several catalytic domains, carbohydrate binding modules (CBMs), non-catalytic modules such as the serpin (Clocl_3968, which may be involved in protein folding or transport), expansins (Clocl_1298 and Clocl_1862, they reduce the crystallinity of plant material and facilitate enzyme action, more details in chapter Expansins), or domains of unknown function (DUFs) (Artzi et al. 2014). The correlating scanning electron microscopy and three-dimensional (3D) super resolution fluorescence microscopy showed that the cellulosomes are highly colocalized on the bacterial cell surface and organized by a defined hierarchy (Artzi et al. 2018). Composition and abundancy of the cellulosomes strongly depend on the type of the substrate. The amount of cell-bound cellulosomes is considerably higher when the cells grow on insoluble substrates such as microcrystalline cellulose or wheat straw in comparison with the cells grown on cellobiose, presumably due to much higher expression of the scaffoldins able to interact with the cell surface, e.g., ScaC. The higher expression of ScaC would be accompanied with higher expression of ScaB and ScaA since all three scaffoldings are parts of an operon controlled under the same regulation (Artzi et al. 2018), and it would result in amplified abundance of the enzymes in the cell-attached cellulosomes.

*Acetivibrio clariflavus* DSM 19732 was cultivated on three carbon sources: cellobiose (CB), microcrystalline cellulose (MCC), and acid-pretreated switchgrass (SG), and cell-free cellulosome complexes were isolated from each culture and tested on their catalytic activity on cellulose, xylan, and switchgrass (Artzi et al. 2015). The isolated cellulosomes were separated by gel filtration chromatography (HiPrep 26/60 Sephacryl S- 500 HR gel filtration column), and in each case, high-molecular (~ 1200 kDa, CB I, MCC I, SG I) and low molecular (~ 400 kDa, CB II, MCC II, SG II) fractions were obtained. The highest cellulolytic activity (on CMC, PASC and MCC) was achieved with MCC I fraction. For xylan hydrolysis, however, higher degradation activities were observed for CB II and MCC II fractions, which may suggest that low molecular fractions are richer in xylanases. Surprisingly, xylan degradation levels were significantly lower for *A. clariflavus* SG cellulosomes in comparison with CB and MCC cellulosomes, presumably due to significantly lower protein levels produced on SG and lower abundance of some xylanases in both SG-I and SG-II fractions, in particular xylobiohydrolase Clocl_1795 (described in detail in chapter Hemicellulolytic enzymes).

Similar results were obtained by Shinoda et al. (2019) who compared the cellulosomes of *A. clariflavus* DSM 19732 and *A. thermocellus* ATCC 27405 grown on delignified rice straw. *Acetivibrio clariflavus* cellulosome exhibited lower activity on insoluble cellulose and higher activity on hemicellulosic substrates, especially on xylan. Again, the xylanolytic ability was mainly attributed to the low molecular complex of the *A. clariflavus* cellulosome. Both cellulosomes efficiently converted delignified rice straw into soluble sugars (saccharification 43% for *A. clariflavus* and. 37% for *A. thermocellus* after 72 h). The saccharification was significantly increased to ~ 80% by a supplementation with β-glucosidase and β-xylosidase. Despite different substrate specificities of both cellulosomes, their combination showed essentially no synergy in the saccharification of plant biomass (Shinoda et al. 2019).

## *Acetivibrio clariflavus* enzymes involved in carbohydrate degradation

### Enzymes degrading cellulose and other β-glucans

To reveal the key cellulosome enzymes, the cellulosomes from *A. clariflavus* cultivated on CB, MCC, and SG were further analyzed. A total of 72 glycoside hydrolases (GHs) were identified, belonging to 27 known GH families (Artzi et al. 2015). The MCC-derived cellulosome was the most active and richest in proteins. The most abundant protein in CB I cellulosome fraction and one of the most abundant proteins in the other fractions is a putative GH48 exoglucanase (Clocl_4007). In general, the GH48 enzymes are essential for the breakdown of crystalline cellulose and mostly act as reducing end cellobiohydrolases (EC 3.2.1.176). Clocl_4007 shows high similarity to the *A. thermocellus* exoglucanase Cel48S (Cthe_2089) and processive endo-β-1,4-glucanase F (Ccel_0729) from *Ruminiclostridium cellulolyticum* (synonym: *Clostridium cellulolyticum*). They both release cellobiose from the reducing end of cellulose (Kruus et al. 1995; Parsiegla et al. 1998). The second putative GH48 exoglucanase domain of *A. clariflavus* is linked to a putative GH9 endoglucanase and form the bifunctional cellulase Clocl_3038 (GH48-GH9-CMB3-CBM3), which is produced as a noncellulosomal enzyme in low levels (about thousandfold lower than Clocl_4007). Family GH9 contains mostly endo-acting cellulases (EC 3.2.1.4) but also several exo-enzymes acting from non-reducing end releasing either glucose (EC 3.2.1.74) or cellobiose (EC 3.2.1.91). Remaining 12 putative GH9 enzymes were detected in significant quantities in the cellulosomes with the highest expression levels for Clocl_3917 (GH9-CBM4) showing similarity to cellobiohydrolase Cbh9A (Cthe_0413) from *A. thermocellus*. Other GH9 members are also similar to various endoglucanases from *A. thermocellus* and *R. cellulolyticum* (Table 1; Supplemental Table S1). The putative *A. clariflavus* exoglucanase Clocl_4007 (GH48-Doc) and the putative processive endoglucanase Clocl_2225 (GH9-CBM3-Doc) (similar to CelQ (Cthe_0625) from *A. thermocellus*) were later recombinantly produced and shown to work synergistically (Artzi et al. 2016). Putative endoglucanases were also found in GH5 and GH8 families. All four GH5 enzymes (Clocl_1981, Clocl_3932, Clocl_0350, Clocl_1122) were detected in the cellulosomes and based on similarity to *A. thermocellus* enzymes are predicted to be endoglucanases. In opposite to the *A. thermocellus* cellulosome, putative GH8 endoglucanase (Clocl_1055) was not expressed by *A. clariflavus* in significant levels. Except of cellulose, non-specific endo-β-1,4-glucanases commonly hydrolyze also mixed linkage β-1,3-β-1,4-glucan, which is abundant in several cereals, e.g., barley. Based on high sequence similarity to biochemically characterized *A. thermocellus* enzymes (Supplemental Table S1), *A. clariflavu* is presumably armed with at least two specific mixed linkage glucanases, sometimes also called lichenases. One of them is a GH16_21 member Clocl_1811 predicted to cleave the polysaccharide β-1,4-linkage following the β-1,3-linkage, similar to *A. thermocellus* homologue Cthe_0211 (sequence identity 82.2%, Supplemental Table 1). The other mixed linkage glucanase is presumably cellulosomal Clocl_3132 (homologue of Cthe_1472, 67.6% sequence identity) from GH26 family.

**Table 1:** GH, CE, and PL domains of *Acetivibrio clariflavus* DSM 19732

CAZy classification	Gene	Modular organization	Predicted catalytic activity	References
**Cellulose degradation**				
GH48	Clocl_4007	GH48-Doc I	**Exo-β-1,4-glucanase**	(Artzi et al. 2016)
GH48	Clocl_3038	GH48-GH9-CBM3-CBM3	Cellobiohydrolase	
GH9	Clocl_1056	GH9-CBM3-Doc I	Processive endo-β-1,4-glucanase	
GH9	Clocl_1566	GH9-CBM3-CBM3-Doc I	Endo-β-1,4-glucanase	
GH9	Clocl_1567	GH9-CBM3-Doc I	Endo-β-1,4-glucanase	
GH9	Clocl_1806	GH9-CBM3-Doc I	Endo-β-1,4-glucanase	
GH9	Clocl_1864	GH9-CBM3-CBM3-Doc I	Endo-β-1,4-glucanase	
GH9	Clocl_1975	GH9-Doc I	Endo-β-1,4-glucanase	
GH9	Clocl_3001	GH9-CBM3-CBM3-Doc I	Endo-β-1,4-glucanase	
GH9	Clocl_3029	CBM30-GH9-Doc I	Non-specific endoglucanase	
GH9	Clocl_3038	GH48-GH9-CBM3-CBM3	Processive endo-β-1,4-glucanase	
GH9	Clocl_3253	GH9-CBM3-Doc I	Non-processive endo-β-1,4-glucanase	
GH9	Clocl_3255	GH9-Doc I	Endo-β-1,4-glucanase	
GH9	Clocl_3917	CBM4-GH9-Doc I	Cellobiohydrolase	
GH9	Clocl_2225	GH9-CBM3-Doc I	**Endoglucanase**	(Artzi et al. 2016)
GH8	Clocl_1055	GH8-Doc I	Endo-β-1,4-glucanase	
GH5_4	Clocl_0350	GH5_4-Doc I-CE2	Non-specific endoglucanase	
GH5_1	Clocl_1122	GH5_1-Doc I	Endo-β-1,4-glucanase	
GH5_1	Clocl_1981	GH5_1-Doc I	Endo-β-1,4-glucanase	
GH5_1	Clocl_3932	GH5_1-Doc I	Endo-β-1,4-glucanase	
GH1	Clocl_3244	GH1	Presumably β-glucosidase	
GH3	Clocl_2040	GH3	β-glucosidase	
GH3	Clocl_2223	GH3-Fn3	Non-specific β-glucosidase	
GH94	Clocl_0464	GH94	Cellobiose phosphorylase	
GH94	Clocl_0465	GH94	Cellodextrin phosphorylase	
**Xylan and arabinan degradation**				
GH10	Clocl_0045	GH10	Endo-β-1,4-xylanase	
GH10	Clocl_0282	CBM22-CBM22-CBM22-GH10-CBM9-SLH	Endo-β-1,4-xylanase	
GH10	Clocl_1543	CBM22-GH10-DUF-CBM9-CBM9-CBM9-CE0-SLH-SLH-SLH	Endo-β-1,4-xylanase	
GH10	Clocl_2083	GH11-GH10-Doc I	**Endo-β-1,4-xylanase Xyn2083**	(Liu et al. 2019)
GH10	Clocl_2194	CBM22-CBM22-CBM22-GH10-CBM9-CBM9-XDoc	Endo-β-1,4-xylanase	
GH10	Clocl_2435	DUF-DUF-CBM9-CBM9-CE0-CBM9-DUF-GH10	Endo-β-1,4-xylanase	
GH10	Clocl_2441	GH11-CBM6-Doc I-GH10	Endo-β-1,4-xylanase	
GH11	Clocl_1480	GH11-CBM6-Doc I-CE6	Endo-β-1,4-xylanase	
GH11	Clocl_2083	GH11-GH10-Doc I	**Endo-β-1,4-xylanase Xyn2083**	(Liu et al. 2019)
GH11	Clocl_2441	GH11-CBM6-Doc I-GH10	Endo-β-1,4-xylanase	
GH11	Clocl_3103	GH11-CBM6-CBM36	Endo-β-1,4-xylanase	
GH11	Clocl_3361	GH11-CBM6-GH11-Doc I-CBM6-CE4	Endo-β-1,4-xylanase	
GH30_12	Clocl_2746	GH30_12-CBM6-Doc I	**Endoxylanase** ***Ac*Xyn30B**	(Šuchová et al. 2024)
GH30_10	Clocl_1795	GH30_10-Doc I	**Xylobiohydrolase** ***Ac*Xbh30A (*Hc*Xyn30A)**	(Šuchová et al. 2021; Crooks et al. 2021)
GH39	Clocl_2443	GH39	**β-xylosidase**	(Geng et al. 2017)
GH43_1	Clocl_0074	GH43_1	**β-xylosidase**	(Geng et al. 2017)
GH43_22	Clocl_0088	GH43_22-CBM42-Doc I	Presumably α-l-arabinofuranosidase	
GH43_4	Clocl_1869	Doc I-GH43_4	**Endoarabinanase Abn43A**	(Geng et al. 2022)
GH43_16	Clocl_2437	GH43_16-CBM6-Doc I	α-l-arabinofuranosidase	
GH43_35	Clocl_2442	GH43_35	**α-l-arabinofuranosidase Abn43C**	(Geng et al. 2022)
GH43_24	Clocl_2763	GH43_24_CBM13-Doc I	Exo-β-1,3-galactanase	
GH43_26	Clocl_3058	CBM42-GH43_26-Doc I	**Exoarabinanase Abn43B**	(Geng et al. 2022)
GH51_1	Clocl_2445	GH51_1	**α-l-arabinofuranosidase Abf51**	(Geng et al. 2019)
GH67	Clocl_0930	GH67	α-glucuronidase	
CE3	Clocl_1893	Doc I-CE3	Acetylxylan esterase	
CE3	Clocl_2097	CE3-DUF-CE3-Doc I	Acetylxylan esterase	
CE3	Clocl_2438	CE3-Doc I	Acetylxylan esterase	
CE4	Clocl_3361	GH11-CBM6-GH11-Doc I-CBM6-CE4	Acetylxylan esterase/chitooligosaccharide deacetylase	
CE4	Clocl_4159	CE4	Acetylxylan esterase	
CE6	Clocl_1480	GH11-CBM6-Doc I-CE6	Acetylxylan esterase	
CE7	Clocl_2221	CE7	Acetyl xylan esterase/cephalosporin-C deacetylase	
CE NC	Clocl_1543	CBM22-GH10-DUF-CBM9-CBM9-CBM9-CE0-SLH-SLH-SLH	Feruloyl esterase	
CE NC	Clocl_2447	CE0-CBM6-Doc I	Feruloyl esterase	
**Mannan degradation**				
GH26	Clocl_0901	CBM35-GH26-Doc I	Endo-β-1,4-mannanase	
GH26	Clocl_1824	CBM35-GH26-Doc I	Endo-β-1,4-mannanase	
GH26	Clocl_3132	GH26-Doc I	Lichenase	
GH130_9	Clocl_1327	GH130_9	β-mannoside phosphorylase	
GH4	Clocl_2639	GH4	α-galactosidase	
**Xyloglucan degradation**				
GH44	Clocl_1564	GH44-Doc I-DUF-CBM44	Non-specific endoglucanase	
GH74	Clocl_0906	GH74-Doc I	Processive exo-xyloglucanase	
GH95	Clocl_2638	GH95	α-l-fucosidase	
**Starch degradation**				
GH13_20	Clocl_2020	CBM34-GH13_20	Cyclodextrinase	
GH13_8	Clocl_3394	CBM48-GH13_8	Unknown	
GH15	Clocl_0563	GH15	Glucoamylase	
**Chitin degradation**				
GH18	Clocl_0081	DUF-GH18	Unknown	
GH18	Clocl_0533	CBM50-CBM50-DUF-GH18	Maybe β-N-acetylglucosaminidase	
GH18	Clocl_3239	GH18-Doc I	Presumably endochitinase	
GH18	Clocl_3662	GH18-CBM	Unknown	
GH18	Clocl_3703	GH18-CBM	Unknown	
GH18	Clocl_3914	DUF-GH18	Unknown	
**Pectin degradation**				
PL9_1	Clocl_2488	Doc I-PL9_1	Pectin/pectate lyase	
PL9_2	Clocl_3954	PL9_2-Doc	Unknown	
PL11_1	Clocl_4161	Doc I-CBM35-PL11_1	Rhamnogalacturonan lyase	
CE8	Clocl_0923	Doc I-CBM35-CE8	Pectin methyl esterase	
CE12	Clocl_3327	DUF-CE12-Doc I-CBM35-CE12	Rhamnogalacturonan acetyl esterase	
**Others**				
GH16_21	Clocl_1811	GH16_21-Doc I	Endo-β-1,3-1,4-glucanase/lichenase	
GH23	Clocl_0677	GH23	Muramidase/lytic transglycosylase	
GH23	Clocl_1565	DUF-GH23	Unknown	
GH23	Clocl_2643	GH23	Unknown	
GH53	Clocl_4012	GH53-Doc I	Endo-β-1,4-galactanase	
GH81	Clocl_1863	DUF-GH81-Doc I	Endo-β-1,3-glucanase (laminarinase)	
GH94	Clocl_3028	GT84-GH94-GH189	Phosphorylase	
GH189	Clocl_3028	GT84-GH94-GH189	β-1,2-glucooligosaccharide-β-glucosyltransferase	
CE2	Clocl_0350	GH5_4-Doc I-CE2	6-*O*-glycoside deacetylase	
CE4	Clocl_1428	CE4	Presumably peptidoglycan N-acetylmuramic acid deacetylase	
CE4	Clocl_2580	CE4	Unknown	
CE NC	Clocl_1551	CE0-Doc I	Unknown	
CE NC	Clocl_2435	DUF-DUF-CBM9_1-CBM9_1-CE0-CBM9_1-DUF-GH10	Unknown	
CE NC	Clocl_3953	CBM3-DUF-CE0-CE0	Unknown	

Enzymes in bold were characterized

More information about enzymes can be found in Supplemental Table S1

*GH*, glycoside hydrolase; *CE*, carbohydrate esterase; *PL*, polysaccharide lyase, *CBM*, carbohydrate binding module; *Doc*, dockerin domain, *XDoc*, dockerin domain binding type II cohesins; *DUF*, domain of unknown function; *GT*, glycosyltransferase; *Fn*, fibronectin domain; *SLH*, S-layer homology domain

For a complete degradation of β-glucooligosaccharides to glucose, β-glucosidase activity is required. Putative β-glucosidases in *A. clariflavus* were found in the families GH1 and GH3. While GH3 enzymes Clocl_2223 and Clocl_2040 are similar to β-glucosidase bglB (Cthe_1256) from *A. thermocellus* and β-glucosidase from *Cellvibrio japonicus*, respectively, the GH1 (Clocl_3244) protein shows only low homology with β-glucosidases (Supplemental Table S1). All three enzymes are non-cellulosomal (do not contain Doc domain). The lack of β-glucosidase (and β-xylosidase) activity in cellulosome was reported by Shinoda et al. (2019) who showed that a supplementation of *A. clariflavus* cellulosome with β-glucosidase and β-xylosidase significantly improved saccharification of delignified rice straw. Moreover, Clocl_2223 and Clocl_3244 are putative intracellular enzymes (they do not contain an obvious signal peptide), which suggests that cellobiose or higher cellooligosaccharides are imported into the cell where they can be processed either by β-glucosidases or by GH94 cellobiose/cellodextrin phosphorylases (Clocl_0464, Clocl_0465) which are also predicted to be intracellular.

### Hemicellulolytic enzymes

Due to the complex structure of the main hemicellulose, xylan, its complete degradation requires a cooperation of several enzymes. Cleavage of the main chain by endo-acting xylanases and exo-acting β-xylosidases must be accompanied by the action of a range of xylanolytic debranching enzymes including α-l-arabinofuranosidases, α-glucuronidases and carbohydrate esterases (CEs) removing acetyl and feruloyl groups. Among xylanolytic enzymes, GH30 enzymes were highly abundant in the *A. clariflavus* cellulosome. A putative GH30 xylanase Clocl_1795 (GH30-Doc) was the second most prominent enzyme in the CB I fraction and one of the most abundant enzymes in all fractions (Artzi et al. 2015). Later, Clocl_1795 was identified as a xylobiohydrolase *Ac*Xbh30A releasing xylobiose from the non-reducing end of xylooligosaccharides and xylans (Šuchová et al. 2021; Crooks et al. 2021). It was a founding member of GH30_10 subfamily. Its crystal structure in complex with xylobiose gave a rationale for its exo-activity and product specificity (St John et al. 2022). Thermophilicity, thermostability, and capability to produce xylobiose from wide variety of xylan polymers make *Ac*Xbh30A a promising candidate for industrial bioprocesses (Singh et al. 2024). Another highly abundant GH30 enzyme (Clocl_2746) bears CBM6 and dockerin modules. Recently, this founding member of the GH30_12 subfamily was characterized as a non-specific endoxylanase showing similar activity on glucuronoxylan and arabinoxylan (Šuchová et al. 2024).

In addition to the GH30 xylanases, several genes coding for putative GH11 and GH10 xylanases were identified in the genome (Table 1). An enzyme bearing xylanase domains from both families (Clocl_2441, GH11-CBM6-Doc-GH10) was upregulated on cellobiose (the CB II fraction) (Artzi et al. 2015). Another GH10 + GH11 protein (designated as Xyn2083, GH11-GH10-Doc) encoded by the gene *clocl_2083* was heterologously expressed in full length as well as individual xylanase domains and characterized (Liu et al. 2019). From glucuronoxylan, the GH11 domain liberated xylobiose and xylotriose, which remained in the hydrolysate. The full-length enzyme GH10 + GH11 initially hydrolyzed the polysaccharide to xylose, xylobiose, and xylotriose, although later xylotriose was completely degraded due to the presence of the GH10 domain. However, catalytic efficiency (*k*_cat_/*K*_*m*_) of the GH11 domain was 71 times higher than that of the GH10 domain. The GH11 domain thus endues Xyn2083 with high catalytic activity and GH10 increases degradation efficiency. An addition of the GH11 domain to cellulase improved hydrolysis of cellulose and xylan in pretreated corn cobs. The degradation of xylan resulted in an exposure of the crystal cellulose, which became accessible to the action of cellulase. Unusually, large GH10 enzymes, Clocl_2194 (CBM22-CBM22-CBM22-GH10-CBM9-CBM9-XDoc) and Clocl_1543 (CBM22-GH10-CBM9-CBM9-CBM9-CE1-SLH-SLH-SLH), were also identified in large quantities. While Clocl_2194 was abundant when the cells were grown on all three substrates, Clocl_1543 was found only in the fractions MCC II and SG II. A homolog of the former enzyme is present in the genome of *A. cellulolyticus*, but absent in *A. thermocellus*, while genes coding for the SLH-containing GH10 enzymes are found in both genomes.

β-Xylosidases are commonly found in GH3, GH39, GH43, and GH52 families. *Acetivibrio clariflavus* lacks GH52 member, and a putative GH3 β-xylosidase is present in the strain 4-2a but not in the type strain DSM19732. GH39 member Clocl_2443 showed relatively high activity towards 4-nitrophenyl β-d-xylopyranoside, but only low activity on xylobiose, while GH43_1 member Clocl_0074 named Xyl43C showed high activity on xylobiose (76.6 U/mg) and efficiently hydrolyzed also higher XOs (Geng et al. 2017). It exhibited relatively high tolerance to xylose, having IC_50_ value of approximately 100 mM.

A complete breakdown of glucurono(arabino)xylan requires an action of α-glucuronidases, which are classified in GH67 and GH115 families. While GH115 α-glucuronidases act on the polymers and are able to debranch internally glucuronylated xylopyranosyl residues, GH67 enzymes remove (4-*O*-methyl-)α-d-glucuronic side chains only from the non-reducing end of glucuronoxylooligosaccharides. Only one gene *clocl_0930* coding for a putative GH67 α-glucuronidase is present in *A. clariflavus* genome. The deduced amino acid sequence does not contain dockerin domain and signal peptide suggesting that the enzyme is non-cellulosomal and intracellular and may be involved in the hydrolysis of glucuronoxylooligosaccharides transported into the cell. Interestingly, GH67 enzymes are not present in *A. thermocellus* and *A*. *cellulolyticus*, but one gene coding for a putative GH67 α-glucuronidase is present in *R. cellulolyticum* genome (Table 2). This may be related to the fact that *A. thermocellus* and *A. cellulolyticus* are not able to ferment xylose, so they do not need to be enzymatically equipped for hydrolysis of glucuronoxylooligosaccharides to monomeric units. None of the four bacteria codes for a putative GH115 α-glucuronidase.

**Table 2:** Distribution of modules of carbohydrate active enzymes in *Acetivibrio clariflavus* DSM 19732, *A. cellulolyticus* CD2, *A. thermocellus* ATCC 27405, and *Ruminiclostridium cellulolyticum* H10

**GH families**	**1**	**2**	**3**	**4**	**5**	**8**	**9**	**10**	**11**	**13**	**15**	**16**	**18**	**23**	**26**	**27**	**30**	**31**	**39**	**42**	**43**	**44**	**48**
***A. clariflavus***^**1**^																							
Genome	1	—	2	1	4	1	13	7	6	2	1	1	6	3	3	—	2	—	1	—	7	1	2
Doc-containing	—	—	—		4	1	12	3	5	—	—	1	1	—	3	—	2	—	—	—	5	1	1
***A. cellulolyticus***^**2**^																							
Genome	2	1	3	—	16	4	21	4	1	2	1	1	5	2	5	—	3	—	—	—	6	1	2
Doc-containing	—	1	—	—	14	3	19	4	1	—	—	1	—	—	5	—	3	—	—	—	6	1	1
***A. thermocellus***^**2**^																							
Genome	2	1	2	—	10	1	16	5	1	2	1	2	4	2	3	—	2	—	1	—	6	1	2
Doc-containing	—	1	—	—	8	1	15	4	1	—	—	1	1	—	3	—	2	—	1	—	6	1	1
***R. cellulolyticum***^**3**^																							
Genome	1	5	4	2	7	3	13	5	2	1	1	—	5	2	2	2	2	2	1	1	10	1	1
Doc-containing	—	1	—	—	6	2	12	2	1	—	—	—	1	—	2	2	2	—	—	—	4	1	1
**GH families**	**51**	**53**	**59**	**62**	**65**	**67**	**73**	**74**	**77**	**81**	**94**	**95**	**105**	**116**	**120**	**124**	**126**	**130**	**133**	**141**	**146**	**189**	**Total**
***A. clariflavus***^**1**^																							
Genome	1	1	—	—	—	1	—	1	—	1	3	1	—	—	—	—	—	1	—	—	—	1	75
Doc-containing	—	1	—	—	—	—	—	1	—	1	—	—	—	—	—	—	—	—	—	—	—	—	42
***A. cellulolyticus***^**2**^																							
Genome	—	1	1	—	—	—	—	1	1	1	3	—	1	1	—	1	—	—	1	1	—	1	94
Doc-containing	—	1	1	—	—	—	—	1	—	1	—	—	1	1	—	1	—	—	—	1	—	—	67
***A. thermocellus***^**2**^																							
Genome	1	1	—	—	—	—	—	1	—	1	3	—	—	—	—	1	1	1	—	1	—	1	76
Doc-containing	—	1	—	—	—	—	—	1	—	1	—	—	—	—	—	1	—	—	—	1	—	—	51
***R. cellulolyticum***^**3**^																							
Genome	3	1	1	2	1	1	1	1	—	—	5	2	—	—	1	—	—	1	1	2	2	1	99
Doc-containing	—	1	1	2	—	—	—	1	—	—	—	1	—	—	—	—	—	—	—	1	1	—	45
**CE families**	**1**	**2**	**3**	**4**	**6**	**7**	**8**	**9**	**12**	**20**	**NC**	**Total**				**PL families**			**1**	**9**	**10**	**11**	**Total**
***A. clariflavus***^**1**^																***A. clariflavus***^**1**^							
Genome	—	1	4	4	1	1	1	—	2	—	6	20				Genome			—	2	—	1	3
Doc-containing	—	1	4	1	1	—	1	—	2	—	2	12				Doc-containing			—	2	—	1	3
***A. cellulolyticus***^**2**^																***A. cellulolyticus***^**2**^							
Genome	1	1	3	4	1	—	1	1	6	1	3	22				Genome			1	1	—	3	5
Doc-containing	1	1	3	1	1	—	1	—	4	1	3	16				Doc-containing			1	1	—	3	5
***A. thermocellus***^**2**^																***A. thermocellus***^**2**^							
Genome	—	1	2	3	—	1	1	1	2	—	4	15				Genome			2	1	—	1	4
Doc-containing	—	1	2	1	—	—	1	—	2	—	4	11				Doc-containing			2	1	—	1	4
***R. cellulolyticum***^**3**^																***R. cellulolyticum***^**3**^							
Genome	1	1	1	4	1	1	1	—	—	1	2	13				Genome			1	—	1	2	4
Doc-containing	—	1	1	1	1	—	1	—	—	—	1	6				Doc-containing			1	—	1	2	4

Data were taken from – 1 – Artzi et al, 2015; 2 – Dassa et al. 2012; 3 – Xu et al. 2013. All data were updated according to CAZy database (www.cazy.org, Drula et al. 2022)

Removal of acetyl groups from the xylan backbone is done by acetylxylan esterases. In *A. clariflavus*, putative enzymes of this specificity were found in families CE3 (Clocl_1893, Clocl_2097, Clocl_2438), CE4 (Clocl_2580_Clocl_3361, Clocl_4159), CE6 (Clocl_1480), and maybe CE7 (Clocl_2221) (Table 1; Supplemental Table S1). Among esterases not classified into a CE family, putative feruloyl esterases (Clocl_1543 and Clocl_2447) were identified.

α-l-Arabinofuranosidases classified in GH43, GH51, GH54, and GH62 families remove arabinose moieties from arabino(glucurono)xylans found in herbaceous plants. Genome of *A. clariflavus* lacks genes coding for GH62 and GH54 enzymes but possesses GH51_1 gene *clocl_2445*, which was cloned, expressed, and the encoded enzyme Abf51 was characterized (Geng et al. 2019). Interestingly, Abf51 is a non-cellulosomal enzyme sharing only 29% identity with the GH51 arabinofuranosidase from *A. thermocellus* (Cthe_2548). The enzyme preferentially cleaved α-1,3-glycosidic bonds, and the hydrolysis of doubly substituted oligosaccharide was much slower. Abf51 significantly increased the conversion of arabinoxylan by GH11 xylanase and a β-xylosidase. The debranching of arabinoxylan and in particular singly decorated arabinoxylooligosaccharides was also demonstrated for non-cellulosomal intracellular GH43_35 Abn43C (Clocl_2442) (Geng et al. 2022). In addition, there are two other putative GH43 enzymes tentatively capable of debranching arabinoxylan: Clocl_0088 (GH43_22) and Clocl_2437 (GH43_16) (Table 1; Supplemental Table S1).

In addition to arabinoxylan-related substrates, Abn43C was also shown to hydrolyze arabinan and branched arabinooligosaccharides. Another two genes (*clocl_1869, clocl_3058*) were found to code for GH43 enzymes active on arabinan (Geng et al. 2022). Abn43A (Clocl*_*1869) (GH43_4) is a unique endoarabinanase producing arabinooligosaccharides and having two times higher activity on decorated sugar beet arabinan (116.8 U/mg) than on linear arabinan. Abn43B (Clocl_3058) (GH43_26) is a specific exo-α-1,5-l-arabinanase releasing arabinose from the non-reducing end of linear arabinan. These three GH43 enzymes worked in synergy during the hydrolysis of arabinan (Geng et al. 2022). The arabinose production from linear arabinan by Abn43B was not affected by Abn43C but increased by 150% by addition of Abn43A, reaching 0.44 g/g arabinan. On the other hand, the efficient hydrolysis of branched sugar beet arabinan requires the action of all three enzymes. An addition of Abn43C to Abn43A and Abn43B boosted the arabinose production 15 times, reaching 0.262 g/g arabinan. The endoarabinanase Abn43A initially cut the main chain, which facilitates the side chains removal by Abn43C and arabinose release by Abn43B (Geng et al. 2022).

Another hemicellulose that is abundant in primary cell walls of dicotyledonous plants is xyloglucan. It is β-1,4-glucan, which is regularly glycosylated at position 6 with α-xylose moieties, which may be optionally further substituted with β-d-galactose or 2-*O*-(α-l-fucopyranosyl)-β-d-galactose disaccharide. A genome of *A. clariflavus* harbors several genes coding for enzymes presumably capable of xyloglucan main chain hydrolysis. These include not only GH74 processive exo-xyloglucanase (Clocl_0906) and GH44 non-specific endoglucanase (Clocl_1564) but likely also some endoglucanases from GH families 5 and 9. Although xyloglucan may be depolymerized, it seems that the derived oligosaccharides are not metabolized further due to a lack of putative α-xylosidase (grouped to GH31 family) and β-galactosidase (mostly in GH35, GH42 and GH2 families). Just putative GH95 α-l-fucosidase (Clocl_2638) can be involved in xyloglucan oligosaccharide trimming.

Degradation of major softwood hemicellulose, galactoglucomannan, is done by endo-β-1,4-mannanases, β-mannosidases, glucanases, and α-galactosidases. *Acetivibrio clariflavus* is predicted to possess two GH26 cellulosomal endo-β-1,4-mannanases Clocl_0901 and Clocl_1824 (Table 1), the former being expressed in higher levels on cellulose and switchgrass. Both are homologous to *A. thermocellus* endo-β-1,4-mannanases Cthe_0032 and Cthe_2811, respectively. Surprisingly, no gene coding for β-mannosidase (GH1 and GH2 families) was found in *A. clariflavus* genome. However, a putative intracellular GH130_9 β-mannoside phosphorylase (Clocl_1327) can substitute its function, resulting in the phosphorolytic degradation of mannooligosaccharides released by endo-β-1,4-mannanases. Removal of α-linked galactose from galactomannan/galactoglucomannan-derived oligosaccharides galactosylated at the non-reducing end can be done by putative GH4 α-galactosidase (Clocl_2639).

### Other carbohydrate active enzymes

The other putative GHs include enzymes for α-glucan conversion from GH families 13 and 15. Their total number of three is in a sharp contrast with the multiplicity of enzymes targeting cellulose or xylan. The limited number of potential α-glucan-modifying enzymes is in line with the inability of *A. clariflavus* to utilize starch as a carbon source (Shiratori et al. 2009), although GH13_20 member (Clocl_2020) may work as endo-acting enzyme, GH15 representative (Clocl_0563) as exo-acting enzyme and GH13_8 catalyst (Clocl_3394) as α-1,4-glucan branching enzyme synthesizing α-1,6-linkages. The organism is not equipped with a phosphorolytic enzyme acting on starch, generating either glucose-1-phosphate (GT35) or maltose-1-phosphate (GH13_3).

Chitin also seems to be degraded only partially. This GlcNAc-based polysaccharide may be hydrolyzed by a putative cellulosomal GH18 chitinase Clocl_3239 (a homologue of *A. thermocellus* chitinase A, Cthe_0270) and putative β-N-acetylglucosaminidase Clocl_0533. The homology of other putative GH18 enzymes to so far characterized enzymes is too low to predict their activities (identity below 20%). Similar is true for putative enzymes from GH23 family, which contains muramidases and lytic transglycosylases (Table 1; Supplemental Table S1). In deacetylation of chitin and peptidoglycan GlcNAc residues, several CE4 enzymes may be involved including Clocl_3361 and Clocl_1428 (Table 1).

There are just two enzymes, Clocl_2763 (putative exo-β-1,3-galactanase from GH43_24 family) and Clocl_4012 (putative GH53 endo-β-1,4-galactanase), that may be involved in β-galactans degradation. Clocl_2763 is a homolog (identity 71.7%) of exo-β-1,3-galactanase from *A. thermocellus* (Cthe_0661), which was shown to release galactose from β-1,3-galactan and β-1,3-galactooligosaccharides, and galactose or 6-substituted galactooligosaccharides from arabinogalactan proteins (Ichinose et al. 2006). Clocl_4012, similar to endo-β-1,4-galactanase Cthe_1400 from *A. thermocellus* (65.9% identity), may have a potential to convert β-1,4-galactans to a mixture of disaccharide and trisaccharide (González-Ayón et al. 2019). However, *A. clariflavus* lacks a canonical β-galactosidase (usually found in GH35, GH42 and GH2 families) that would cleave the galactooligosaccharides to galactose. Therefore, it seems that primary role of Clocl_2763 and Clocl_4012 is not to generate monosaccharide for further catabolism but rather partially cleave β-galactans to make other cell wall polysaccharides more accessible for other enzymes.

*Acetivibrio clariflavus* is poorly equipped with the enzymes involved in the breakdown of pectic substances. There is no endo- or exo-acting glycoside hydrolase active on homogalacturonan (GH28 family). This acidic polysaccharide may be attacked by two putative PL9 lyases Clocl_2488 (PL9_1) and Clocl_3954 (PL9_2) presumably having preference for pectin (methyl esterified from) and pectate (non-esterified from), respectively. The deesterification may be catalyzed by Clocl_0923 that is a putative pectin methyl esterase from CE8 family. Similarly, rhamnogalacturonan may be attacked only by a putative rhamnogalacturonan lyase Clocl_4161 from PL11 family and a putative rhamnogalacturonan acetyl esterase Clocl_3327 from CE12 family due to a lack of α-L-rhamnosidase (classified into GH78, GH90, and GH106 families) and α-galacturonidase (GH28). However, the fragments generated by the PL9 and PL11 lyases, which comprise 4,5-unsaturated α-d-galacturonic acid residue on the newly formed non-reducing end, seemingly remain intact due to a lack of the corresponding 4,5-unsataurated uronic acid hydrolase from GH105 family.

## Carbohydrate binding modules

In addition to the catalytic domains, the enzymes acting on polysaccharides often contain carbohydrate binding modules (CBMs), which serve to bring the enzymes to a close proximity of the substrate. In total, 83 CBMs from 19 different CBM families were identified in the proteins from *A. clariflavus* (Table 3). The highest number (19) is from the family CBM3, sometimes organized in a dyad attached to a C-terminus of the catalytic domain. In this arrangement (catalytic domain-CBM3-CBM3), the first CBM3 module is usually functionally linked to the catalytic domain, while the C-terminal CBM3 module anchors the protein to a polysaccharide. The members of the CBM3 family are known to bind a cellulose, and in *A. clariflavus*, they are mostly connected to cellulolytic GH9 catalytic domains. However, novel CBM3 modules were discovered in *A. thermocellus* and *A. clariflavus* (Cthe_0271 and Clocl_1192), which exhibited binding to xylan rather than to cellulose (Rimon et al. 2021). The loss of the cellulose-binding ability in both proteins is caused by a lack of several aromatic amino acid residues in the area of cellulose-binding strips. *Acetivibrio clariflavus* GH9 domains are also linked to CBM4 and CBM30 modules, which might bind cellulose, glucans, and xylan (Table 3). Twelve CBM6 modules binding cellulose, glucans and xylan are present in eight proteins, which mostly contain also xylanolytic domains from GH11 (4), GH10 (1), GH43 (1), and GH30 (1) families. CBM9s (nine in total) are always found with GH10 domains. Seven CBM9s are present in three large multidomain GH10 xylanases together with CBM22s (seven in total). Similar multidomain enzymes with CBM9-GH10-CBM22 motif are found also in other bacteria, e.g., *A. thermocellus* and *Caldicellulosiruptor acetigenus*, in which the functions of CBM9s and CBM22s were tested (Selvaraj et al. 2010; Krska and Larsbrink 2020; Krska et al. 2021). It was shown that both types of domains bind insoluble cellulose and beech and birch xylan, but some of them are also able to bind ivory nut mannan, xyloglucan, or wheat arabinoxylan. It seems that their binding preferences complement each other to ensure maximal approximation of catalytic domains to complex cell wall polysaccharides for efficient catalysis. CBM22s are also considered as thermostabilizing domains, so they may have a dual function. CBM2s (4), which are known to interact with either cellulosic substrates or xylans, are parts of non-catalytic scaffoldins. It is interesting that CBM2s are not present in proteins from *A. thermocellus*, *A. cellulolyticus*, and *R. cellulolyticum*.

**Table 3:** CBMs in *Acetivibrio clariflavus* DSM 19732

CBM	Number of CBMs^*^	Putative functional domain(s) attached	Ligand(s) identified for CBM family
**Cellulolytic activity**			
CBM3	19 (15)	9 attached to GH9 domain, 4 to DUFs, 1 is connected to DUF as well as CE4-like domain and 1 is a part of scaffoldin ScaA	Cellulose
CBM4	1	GH9 (cellobiohydrolase)	Xylan, β-1,3-glucan, β-1,3-1,4-glucan, β-1,6-glucan and amorphous but not crystalline cellulose
CBM30	1	GH9	Cellulose and beta-1,3-1,4-mixed glucan
**Xylanolytic activity**			
CBM6	12 (8)	Attached to xylanolytic enzymes from GH11 (4), GH10 (1), GH43 (1), GH30 (1), CE4 (1), CE6 (1) and to DUF (1)	Amorphous cellulose, β-1,4-xylan, β-1,3-glucan, β-1,3-1,4-glucan, and β-1,4-glucan
CBM9	9 (4)	Present in several copies in large proteins together with CMB22. All 4 proteins contain GH10 domain, 2 of them also CE0 and 1 also DUF.	Cellulose, xylan, mannan
CBM22	7 (3)	Always together with CBM9 and GH10	Xylan and mixed β-1,3-1,4-glucans
CBM36	1	GH11	Xylan
CBM13	1	GH43_24	Xylan, galactose, arabinose
CBM42	3	GH43_22, GH43_26, DUF	Arabinofuranose (present in arabinoxylan)
**Starch hydrolysis**			
CBM34	1	GH13_20	Starch
CBM48	1	GH13_8	Starch oligosaccharides
**Other**			
CBM35	6	GH26, CE12, CE8, PL11	Xylan, mannan, β-galactan
CBM16	3	GT39	Cellulose and glucomannan
CBM50	9 (7)	All are connected to DUFs and in one case also to GH18	Chitin
**No catalytic activity**			
CBM2	4	Parts of scaffoldins	Cellulose, chitin or xylan
CBM63	2	Expansins	Cellulose
**DUF**			
CBM32	1		Galactose and lactose, polygalacturonic acid, N-acetyllactosamine
CBM44	1		Cellulose and xyloglucan
CBM54	1		Xylan, yeast cell wall glucan and chitin
CBM_NC	7		

*The values in parentheses indicate the number of proteins containing given CBM module

## Expansins

Among dockerin-containing proteins of *A. clariflavus*, two expansin-like proteins, Clocl_1862 and Clocl_1298 (*Ccl*EXL1 and *Ccl*EXL2, respectively), were identified (Artzi et al. 2016; Chen et al. 2016). Expansins are small non-catalytic proteins (~ 26 kDa) with disruptive activity on plant cell walls. They interfere with non-covalent interactions between the plant cell wall polysaccharides, thus loosening the crystallinity of the complex substrate by physical separation of the associated polysaccharide chains, which makes the plant cell wall polysaccharides more accessible to the degrading enzymes. It was shown that the expansin-like protein *Ccl*EXL1 recognized preferentially cellulosic substrates, interacting stronger with microcrystalline cellulose than acid-swollen cellulose, but it also partially bound to xylan and wheat straw. *Ccl*EXL1-mediated enhancement of filter paper and microcrystalline cellulose degradation was demonstrated for different cellulosome fractions as well as two major cellulosomal GH48 and GH9 cellulases (Artzi et al. 2016; Chen et al. 2016). The enhancing effect of *Ccl*EXL2 was even stronger, being exhibited not only with native *A. clariflavus* cellulosomes but also with an artificial minicellulosome (Chen et al. 2016). However, the mechanism of expansin action is complex and still not completely understood. Their contribution to biomass degradation depends on a variety of factors such as type of enzymes and substrates used, the ratios between the expansin and the enzymes, and timing of expansin addition to the substrate. It was demonstrated that the preincubation of the substrates with *Ccl*EXL1 before adding the cellulases is important when working with artificial enzyme cocktails (Artzi et al. 2016). Generally, the cellulosomal expansin-like proteins are important in lignocellulose degradation by *A. clariflavus* and they could be used to enhance cellulosome efficiency in lignocellulose bioconversion (Chen et al. 2016).

## Conclusions

The thermophilic bacterium *A. clariflavus* is equipped with a wide range of cellulolytic and hemicellulolytic enzymes. Many of them are arranged in cellulosomes where these enzymes are in close proximity to each other as well as with their target polysaccharides, resulting in synergistic action and efficient degradation of plant material. Twenty-five putative enzymes are predicted to be involved in cellulose degradation, and even more (33) are predicted to be xylanolytic. Despite the wide spectrum of cellulose utilization catalysts, in comparison with other well-known anaerobic cellulosome-forming bacteria *A. thermocellus* and *A*. *cellulolyticus*, *A. clariflavus* has the lowest number of GH9 glucanases and GH5 enzymes, which are usually involved in cellulose breakdown (Table 2). This tentative reduced efficiency of cellulose decomposition is, however, compensated with a higher xylanolytic potential due to significantly increased number and versatility of xylanolytic enzymes including endoxylanases as well as accessory xylanolytic enzymes (e.g., GH67 α-glucuronidase absent in *A. thermocellus* and *A*. *cellulolyticus*). In terms of the number of cellulolytic/xylanolytic enzymes, *A. clariflavus* resembles more *R. cellulolyticum*, which is even better equipped for hemicellulose degradation (Table 2). Moreover, xylose-fermenting ability of *A. clariflavus* 4-2a strain makes it a perfect candidate for highly efficient biomass degradation. The fact that the bacterium is armed with endo-acting glycanases depolymerizing cellulose and mannan as well as with phosphorylases utilizing the derived oligosaccharides paves the way for economically viable generation of glucose-1-phosphate and mannose-1-phosphate as precursors for donors utilized by glycosyl transferases in the synthesis of various glycoconjugates.

## Supplementary Information

Below is the link to the electronic supplementary material.

Supplementary file1 (XLSX 32 KB)

## Data Availability

The data presented in this study are available within the paper and its Supplementary Information files.
